# Innovation in Biomedicine: Can Stem Cell Research Lead the Way to Affordability?

**DOI:** 10.1371/journal.pmed.0030126

**Published:** 2006-02-28

**Authors:** Merrill Goozner

## Abstract

In 2004, California voters approved a US$3 billion stem cell research program. Goozner outlines his innovative suggestions for ensuring that any treatments resulting from the program will be made affordable for state residents.

In November 2004, California voters approved a ten-year, US$3 billion stem cell research program to pursue cures for diabetes, Parkinson disease, spinal cord injuries, and other chronic conditions. Campaign organizers also claimed the state would receive royalties from new therapies, economic development in the form of jobs and taxes, and access to cheaper medicines [1]. Once the initiative passed, its proponents sought to scale back unrealistic voter expectations about rapid advances in the field—recent revelations of scientific fraud involving a prominent stem cell scientist will undoubtedly have that effect.

Yet the goal that stem cell therapies resulting from the initiative will be made affordable for state residents remains in place. Toward that end, some California legislators are focusing on how the newly created California Institute for Regenerative Medicine (CIRM) should handle intellectual property (IP) generated by its grants. In August 2005, at CIRM's request, the state-funded California Council on Science and Technology (CCST) recommended that CIRM adopt with minor variations the federal Bayh–Dole system [2].

The 1980 Bayh–Dole Act gives research institutions the primary responsibility for maximizing the health- and economic-development benefits from government research funding. It encourages researchers or their institutions to patent inventions generated under government grants and transfer the technology to private firms. While the act gives the federal government the power to influence the affordability of the resulting technologies, it has never used this authority. The CCST report, embracing that stance, discouraged efforts to recoup revenue through high licensing fees and postponed a discussion of preferential pricing for state residents [3]. The report suggested such approaches would inevitably hinder the development of the public–private collaborations needed to bring new therapies to market.

While regional governments frequently fund biomedical research in Europe, California is the first state in the United States to embark on a large-scale program. The size of its commitment suggests that the state will be a major patron of stem cell research for years to come. This gives California a unique opportunity to create a climate that will not only be hospitable to innovation but also simultaneously deliver affordable medicine. The state government can do this by redefining how government, medical researchers, and the private sector interact. In doing so, it could serve as a model for reforming the US and global biomedical innovation systems.

Change is necessary for two reasons. First, under the current system, new technologies, no matter how marginally effective, come to market at the highest prices. These advancing medical technologies are a major cause of rapidly rising health-care spending throughout the industrial world. Second, biomedical innovation in the US, long considered the global leader, has slowed markedly in the past half decade. Despite escalating research spending in the public and private sectors, the number of new drugs and biologics recently approved by the US Food and Drug Administration (FDA) has fallen below previous eras (Figures 1 and 2). And those new therapies that have been approved tend to have less significance than medical advances of the past. While the popular press excitedly reports that breakthroughs in nanomedicine, targeted therapeutics, and genomic medicine are just around the corner, applications to launch clinical trials have fallen well below the levels of the early 1990s (Figure 3). Something beyond the usual culprit—higher failure rates—is at work [4].

**Figure 1:**
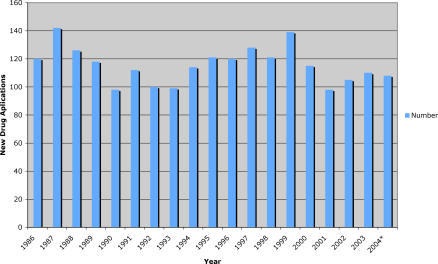
Applications for New Drug Approvals Asterisk indicates that new biologics applications were included in data for the first time. (Source: FDA, http://www.fda.gov/cder/rdmt/numofndareccy.htm)

**Figure 2:**
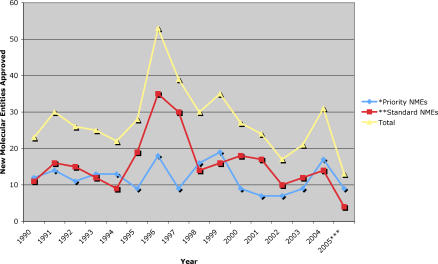
New Drug Approvals at the FDA *Priority NMEs are new molecular entities that represent a significant improvement compared with marketed products in the treatment, diagnosis, or prevention of a disease. **Standard NMEs are new new molecular entities that appear to have therapeutic qualities similar to those of one or more already marketed drugs. ***Data through Nov 30, 2005. Note that in 2004, the FDA began including biologics in with its new drug approval data. These data have been excluded from this chart. In 2004, there were four priority biologics and one standard biologic approved by the FDA; in 2005 through November 30, 2005, there was one priority biologic and no standard biologics approved by the agency.

**Figure 3:**
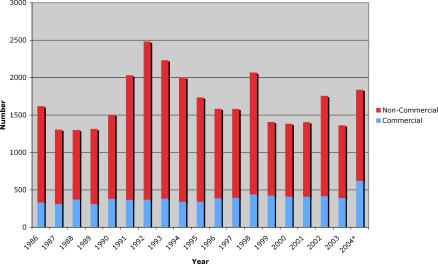
Applications to Begin Human Clinical Trials Asterisk indicates that investigative new drug applications for therapeutic biologic products were included for the first time.

## Patent Thickets

The IP system may be contributing to the slowdown. The current innovation system encourages researchers to patent and commercialize discoveries that in an earlier era were considered basic science insights. This has led to an active market in the building blocks of further research, which can be anything from a genetic sequence or a cell receptor to the reagents needed to culture cells. This proliferation of basic science patents has raised the bar—what economists call transaction costs—for other researchers who want access to those research tools. While many researchers, especially in academia, find ways around patent restrictions, and many companies have no trouble executing license agreements, there are cases where “patent thickets” have discouraged other researchers from pursuing similar or subsequent lines of inquiry [5].

The stem cell field, which is still years away from its first approved therapy, has already experienced patent thicket problems. In May 2005, *Nature* drew attention to the case of Jeanne Loring, an embryologist at the Burnham Institute in La Jolla, California [6]. She claimed her startup firm collapsed when it couldn't get access to embryonic stem cells at a reasonable price from the Wisconsin Alumni Research Foundation, which owns James Thomson's seminal patents in embryonic stem cell research. The Wisconsin Alumni Research Foundation has granted several exclusive licenses to Geron, Inc., which funded his work [7].

A recent survey by the United Kingdom Stem Cell Initiative identified nearly 18,000 stem cell patents issued around the world since 1994, with two-thirds issued in the US [8]. The Washington-based law firm of Sterne Kessler Goldstein and Fox has warned clients that “any company or research institution that plans to develop stem cells for therapeutic purposes may face a number of blocking patents and applications that will require licenses, if available” [9]. The potential for patent licensing restrictions to slow the pace of research is impossible to quantify, but surely exists. How does one count the decisions of researchers who eschew a line of research because they don't want to bother securing the necessary licenses or material-transfer agreements? How does one count the decisions of researchers to avoid fields entirely because someone else has already locked up key inventions? How can one predict if cascading licensing fees will make downstream research prohibitively expensive?

## Jumping into the Pool

CIRM and other stem cell funders can become catalysts for cutting through this patent thicket. They can require that all grant recipients agree to donate the exclusive license to any insights, materials, and technologies that they patent to a common patent pool supervised by a new, nonprofit organization set up for that purpose. A patent pool serves as a one-stop shop where investigators can obtain no-cost or low-cost licenses for subsequent research. Patent pools have been successfully used in other high-technology industries such as consumer electronics and software to facilitate the development of new technologies that either require common standards or rest on a common base of basic research. Several patent law firms and close observers of medical research have suggested that patent pools can work in biomedicine [9,10].

There is already some official interest in the patent pool approach, at least for early stage research. The CCST report to CIRM suggested mechanisms such as broad-use licenses could be used to facilitate the sharing of software, databases, and other research tools (see page 14 in [2]). The UK Stem Cell Initiative, a public–private partnership, included a call for a new UK Stem Cell Cooperative “to maximize the cross-fertilization between those involved in the subdisciplines of UK stem cell research” (see page 8 in [8]).

But the stem cell patent pool needs to reach beyond the early stages of research if it is going to maximize the chances of this targeted research campaign eventually producing therapeutic results. As researchers move further down the development trail, the pool can serve as a clearinghouse for all researchers in the public or private sector to gain permissions for pursuing the next stage of their research at minimal transaction costs, including time. Moreover, the pool authority can act as an agent for resolving challenges that will inevitably arise as the research progresses, including enforcing ethical standards. For instance, the pool authority in cooperation with the FDA can set common standards for cell line preparations as research moves toward the critical clinical trial phase. And the pool should have the scale to leverage the cooperation of existing patent holders whose IP predates formation of the pool or whose future research will be funded by other governments, nonprofits, or private firms.

The pool can also influence accessibility to the fruits of downstream research. As a condition for obtaining a pool license, any researcher would have to contribute any IP that results from using the pool license back into the pool. In the software world, this is known as open-source licensing, which was used successfully to develop the still-evolving Linux computer operating system and which is being pursued in agricultural biotechnology (R. Jefferson, personal communication; [11]).

There is one major stumbling block for the use of an open-source patent pool to facilitate stem cell research. Unlike software or even agriculture biotechnology—where the end products are relatively low cost, and the costs of development are relatively evenly distributed throughout the development process—biomedical research costs escalate once a therapeutically useful product reaches clinical trials. Applied research can take five to ten years from the start of human safety experiments. While the costs of pharmaceutical research are less than the drug industry claims, the investment required can run into the tens or even hundreds of millions of dollars. As a result, this developmental research has almost always been funded by the private sector [12]. (There are, of course, many exceptions to this rule: the early HIV/AIDS medications, many cancer drugs, some vaccines, and the development of several rare-disease therapies have been entirely funded by government agencies.) The private sector's price for taking these late-stage risks is exclusive rights to the technology. Its reward, if successful, is the right to charge whatever the health-care marketplace will bear.

## Eyes on the Prize

However, there is an alternative to the exclusive rights/high prices model used by conventional markets. A government body such as CIRM could establish a major prize for companies and institutions that collaborate to produce a successful stem cell therapy. The prize would have to be large enough to justify the substantial investment required to carry out the final stages of research. It would also have to be large enough to share with the upstream patent holders whose basic and applied research became part of the pool that led to the new therapy. One could imagine prizes in the billions of dollars based on considerations such as the prevalence and public health impact of the disease, the difficulty in developing its cure, and the capital investment required to achieve results. A prize system has been proposed at the federal level [13].

A prize system, coupled with an open-source patent pool, is entirely consistent with the existing IP system. Inventors and their institutions would retain the IP rights to their inventions. Any revenues generated from the prize could be shared with the inventor and reinvested in research and education. Though the rights to the invention would be turned over to the pool, the technology-transfer officials at an institution would still have an incentive (their share of the prize) to aggressively pursue its use by downstream scientists in the public or private sectors if they feel their invention is not being properly utilized.

Division of the prize could be based on mandatory arbitration among patent holders [14]. Or it could be based on the value of the research contracts that led to the underlying IP and were invested in clinical trials. Basing the prize on investment would weight its distribution toward the parties that conducted the final phases of research—usually private-sector firms—since the trials are generally the most expensive part of therapeutic development.

Governments can finance the prizes using tax-exempt bonds since a prize will only be awarded for success. At that point, the bonds can be repaid by a surcharge on each use of the new therapy as it rapidly diffuses through the health-care system. Once the prize has been awarded for a successfully developed stem cell therapy, the pool authority can grant licenses to one or more generic manufacturing firms, which can then compete to sell the therapy to health-care providers and the public on a cost-plus basis [15].

Wouldn't the surcharge to finance the prize, when added to the cost-plus production by generic manufacturers, add up to the same high prices for medicines generated by the current system? Not at all. This “shared prize model,” calibrated to the true cost of research and development, eliminates the 30%–40% of pharmaceutical industry revenue generated by wasteful marketing costs. The prize provides no rewards for industry research and development that goes to develop medicines that duplicate the action of medicines already on the market. Financing the prize with tax-exempt bonds ensures that the surcharge will be based on the lowest-cost capital available.

## Conclusion

By combining a patent pool, an open-source model of IP development, and a shared prize system for developing stem cell therapies, the California state stem cell program can point the way to a new medical innovation system for the 21st century. This model could be used by all advanced industrial economies grappling with how to pay for the rising cost of the new medical technologies sought by their ill and aging populations.

## Abbreviations

CCST: California Council on Science and Technology
CIRM: California Institute for Regenerative Medicine
FDA: US Food and Drug Administration
IP: intellectual property
